# Advances in the development of improved animal-free models for use in breast cancer biomedical research

**DOI:** 10.1007/s12551-017-0276-4

**Published:** 2017-07-26

**Authors:** Sophie Roberts, Valerie Speirs

**Affiliations:** Leeds Institute of Cancer & Pathology, University of Leeds, St James’s University Hospital, Wellcome Trust Brenner Building, Leeds, LS9 7TF UK

**Keywords:** Breast cancer, Ex vivo models, Tissue banks

## Abstract

Through translational research, the outcomes for women (and men) diagnosed with breast cancer have improved significantly, with now over 80% of women surviving for at least 5 years post-diagnosis. Much of this success has been translated from the bench to the bedside using laboratory models. Here, we outline the types of laboratory models that have helped achieve this and discuss new approaches as we move towards animal-free disease modelling.

## Introduction

**Fig. 1:**
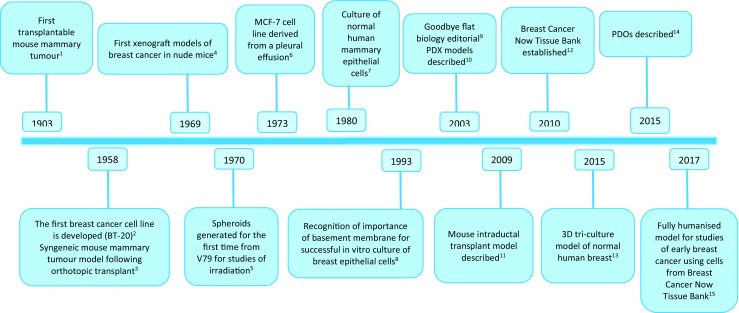
Advancements of breast cancer models over time. The timeline presents the fundamental breakthroughs in breast cancer models over time. ^1^Cardiff and Kenney (2011), ^2^Lasfargues and Ozzello (1958), ^3^Deome et al. (1959), ^4^Rygaard and Povsen (2007), ^5^Sutherland et al. (1970), ^6^Soule et al. (1973), ^7^Stampfer et al. (1980), ^8^Petersen et al. (1992), ^9^Abbott (2003a, b), ^10^Beckhove et al. (2003), ^11^Behbod et al. (2009), ^12^BCN, ^13^Nash et al. (2015), ^14^van de Wetering et al. (2015), ^15^Carter et al. (2017). Image adapted from Holen et al. (2017)

Laboratory models to study breast cancer behaviour and response to therapy have been instrumental in contributing to improving patient outcome. Starting from simple cell culture models using immortalised human cell lines derived from patient tumours grown in two dimensions (2D), these have gradually evolved into more complex three-dimensional (3D) multi-cellular models and, lately, towards patient-derived organoid models (Soule et al. 1973; Wang et al. 2002; Debnath et al. 2003; Nash et al. 2015; Bruna et al. 2016). Animal models have also been employed first using cells lines growing as xenografts (Deome et al. 1959) and, more recently, using so-called patient-derived xenograft (PDX) models (Whittle et al. 2015). The guiding principles for the improved welfare of animals used in research were introduced in 1959 and termed the 3Rs: replacement, reduction and refinement (Russell and Burch 1959). These have been implemented in many countries to support the humane use of animals in laboratory research. There are now specific funding bodies which exclusively support research which either completely replaces (e.g. Animal Free Research UK; https://www.animalfreeresearchuk.org), reduces or refines the use of animals in research (e.g. the National Centre for the Replacement, Refinement & Reductions of Animals in Research in the UK; https://www.nc3rs.org.uk and Medical Advances Without Animals in Australia; http://www.mawa-trust.org.au). Many scientists are now actively engaged in further advancing this ethos, by developing improved scientific methods, which serve to reduce the reliance on animals in biomedical research or to completely replace them. A timeline showing key achievements towards the advancement of breast cancer models in biomedical research is shown in Fig. 1. We discuss the various models available and their pros and cons below.

## Cell lines

Cell lines have been the workhorses in biomedical research labs for decades. The first and arguably the best known is HeLa, a cervical cancer cell line derived from tissue taken from Henrietta Lacks (Gey et al. 1952). The first breast cancer cell line, BT20, was developed in 1958 from an invasive ductal carcinoma (Lasfargues and Ozzello 1958); however, the most commonly used breast cancer cell line in the world is MCF-7, described in 1973 (Soule et al. 1973) and derived from a pleural effusion from an invasive ductal breast cancer which developed in a 69-year-old Caucasian nun, Frances Mallon. Since then, a number of different breast cancer cell lines have been developed, and the latter half of the 20th century allowed scientists to use these through in vitro cell culture or in animal experiments using xenografts, in experiments designed to better understand the biology of breast cancer. This research has helped in the development of new diagnostic tests and new treatments, e.g. the presence of HER2 to determine which patients are likely to derive benefit from trastuzumab and the development of tamoxifen for the treatment of breast cancer (Gottardis et al. 1988; Slamon et al. 1989).

**Fig. 2:**
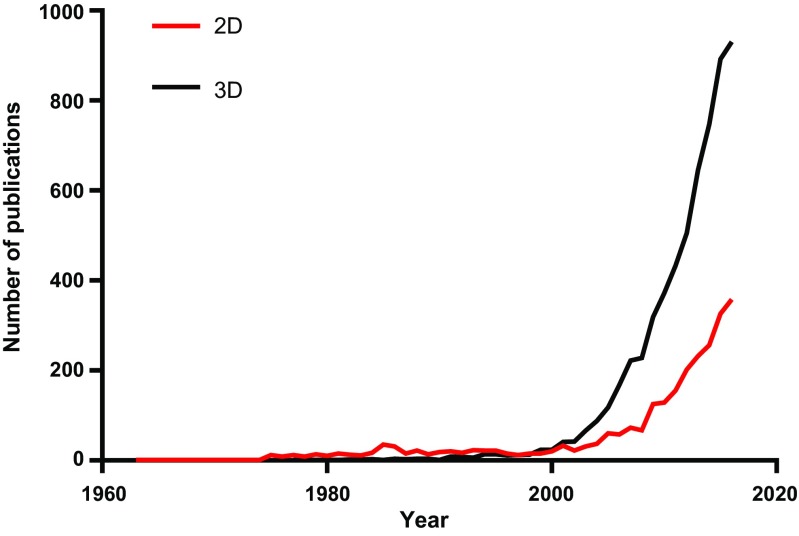
Interrogation of PubMed (2 June 2017) shows that the number of publications reporting 3D cell culture has overtaken that of 2D culture and continues to grow exponentially

While cell lines are convenient research tools to study breast cancer, they are relatively simplistic models, representing a reductionist approach to disease modelling, as they lack the complexity and heterogeneity which characterise human breast tumours. Not only is breast cancer complex with many different subtypes, it know well recognised that the tumour microenvironment can influence breast cancer epithelial cells (Noël and Foidart 1998). Moreover, traditional methods of culturing cells in isolation on plastic substrates further remove this complexity, potentially limiting the translational impact of laboratory findings into the clinic. Given the multi-faceted inter-relationship of cells with their microenvironment in native tumours, scientists have recognised the shortfalls of this reductionist approach, as culturing cells in 2D in tissue culture plastic is not synonymous with this. This was tackled initially in co-culture experiments, where cancer epithelial cells were grown with fibroblasts, the principal cell type within the stromal microenvironment, leading to important insights into how stromal fibroblasts could influence tumour epithelial cells (van Roozendaal et al. 1996; Dong-Le Bourhis et al. 1997; Smith et al. 2015). A News Feature and accompanying Editorial entitled “Goodbye, flat biology?” published in Nature (Abbott 2003a, b) was a rallying call to scientists to consider adopting more relevant 3D models, with due consideration of the microenvironment. This was the first time 2D culture was officially challenged by a high-impact journal. Since then, the number of papers reporting 3D cell culture has overtaken that of 2D culture and continues to grow exponentially (Fig 2).

## 3D culture using cell lines

Three-dimensional spheroids were first generated using Chinese hamster V79 cells growing in spinner flasks to study the effects of irradiation (Sutherland et al. 1970). Since then, the use of spheroids in cancer research has advanced greatly. The classification of a spheroid is poorly defined but is generally thought of as the formation of a rounded 3D structure composed of multiple cells. Spheroids are good models of cancer as they develop pH, hypoxic and proliferative gradients akin to the avascular stages of solid tumours (Nederman et al. 1984; Rotin et al. 1986). This arrangement is mirrored in native tumours, where the outer cells are the only ones with sufficient contact to a blood supply containing nutrients needed for growth.

There are several ways in which breast cancer spheroids have been cultured. Initial approaches involved plating cell suspensions on an agar–base medium as a means of restricting cell–substrate adhesion (Yuhas et al. 1978), the so-called liquid overlay technique. Other cell types, notably fibroblasts, were added (Seidl et al. 2002). Subsequently, the use of low-attachment plastics allowed spheroid formation (Pickl and Ries 2009). The availability of reconstituted basement membrane, Matrigel™, allowed 3D culture of normal and tumourous human breast cell lines (Wang et al. 2002; Debnath et al. 2003; Ivascu and Kubbies 2007) and, in the case of normal, MCF-10A mammary epithelial cells, formed acini-like spheroids that recapitulated facets of the native mammary gland (Debnath et al. 2003). Collagen matrix was also adopted as a means of offering greater physiological relevance (Holliday et al. 2009; Roberts et al. 2016), and a range of natural and synthetic matrices have since been used for 3D culture of breast cancer cells (Bissell and Bilder 2003; Lee et al. 2007; Russ et al. 2012; Nash et al. 2015). Other techniques include the liquid overlay technique (Ivascu and Kubbies 2007) and hanging drop method (Nagelkerke et al. 2013). More recently, our group has used a fully humanised cell culture medium, which encouraged spheroid formation in the absence of supporting matrix (Roberts et al. 2016).

With recognition that the tumour microenvironment plays a pivotal role in cancer formation and progression, spheroid models have become more complex and multi-cellular to reflect this. Multiple cell types are found in the tumour microenvironment, including fibroblasts, macrophages and immune cells, with cancer-associated fibroblasts (CAFs) being the main cell type (Buchsbaum and Oh 2016). As a result, more advanced heterotypic 3D models incorporating the tumour stroma have been generated, e.g. the 3D co-culture of cancer cells with CAFs (Sadlonova et al. 2005; Olsen et al. 2010; Li and Lu 2011; Pinto et al. 2014) and the incorporation of immune cells (Augustine et al. 2015). Such models more closely replicate the tumour environment in vivo. These also include pioneering 3D models of breast cancer metastasis to bone using metastatic breast cancer cell lines seeded onto human subchaodral bone discs (Holen et al. 2015). These types of models are important, as models of cancer metastasis have been limited to animal xenograft models, yet these do not recapitulate the human bone microenvironment.

Nevertheless, spheroids do have their limitations. Different cell types have varying abilities to form spheroids; for example, the BT-474 HER2 overexpressing cell line forms tightly packed rounded spheroids, whereas the SKBR3 HER2 overexpressing cell line forms loose, grape-like aggregates (Froehlich et al. 2016; Roberts et al. 2016). Also, it can be challenging to control the size of spheroids formed and, therefore, the reproducibility of experiments for high-throughput drug screening is limited. This has been examined recently, where 42 different experimental methods were evaluated to test how well spheroid formation was induced using three commonly used breast cancer cell lines; MCF-7, MDA-MB-231 and SKBr3 (Froehlich et al. 2016). Further work in addressing these limitations could make them stronger tools for cancer research in the future.

## Animal models

The significance of using animal models in breast cancer research has recently been reviewed comprehensively (Holen et al. 2017), and the reader is directed to this article for up-to-date information. While there is no doubt that these models have contributed to some of the success in translating laboratory findings to the clinic, they have limitations as pre-clinical models. This is exemplified by the high attrition rates of promising pre-clinical agents when entered into clinical trials (Kola and Landis 2004). Scientists are now applying lateral thought to implement better ways of modelling breast cancer and models developed from human clinical material are starting to gain traction. These are discussed below.

## Primary cell culture

**Fig. 3:**
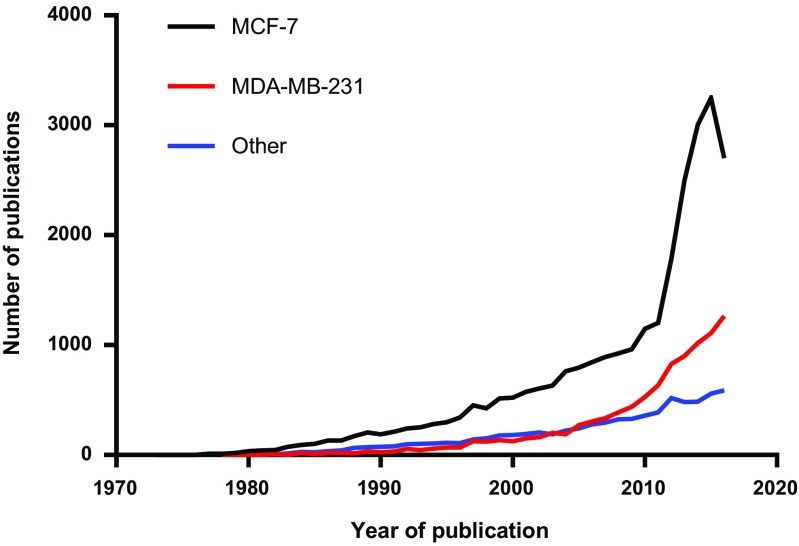
Interrogation of PubMed (2 June 2017) shows that the use of breast cancer cell line MCF-7 far exceeds the use of all other breast cancer cell lines in biomedical research

Recognition that breast cancer was classified into at least four major molecular subgroups (Perou et al. 2000) allowed scientists to reclassify existing cell lines into representative examples (Neve et al. 2006; Holliday and Speirs 2011). However, use of the panel of cell lines available tends to be skewed in favour of the most common Luminal subgroup, exemplified by the ‘workhorse’ of breast cancer research, MCF-7. This is shown in Fig. 3, where the number of papers in PubMed which have used MCF-7 far exceeds those using the second most common breast cancer cell line, MDA-MB-231, often used to model the more aggressive triple negative breast cancer, while aggregate publications of other less commonly used breast cancer cell lines, e.g. to represent HER2-positive breast cancer, is lower still. This has led scientists to consider alternative models using human clinical material.

Generating primary cells from tissue biopsies or resections is regarded by many as a step up from cell lines, moving towards achieving greater clinical relevance in biomedical research. Primary cell culture is challenging, at least in breast cancer, where, paradoxically, it is often easier to generate normal epithelial cells than cancer cells (Wang et al. 2002). Furthermore, overgrowth by fibroblasts is a perennial problem. Hence, a degree of skill and perseverance is required to achieve this successfully. Nevertheless, this has been achieved by a number of groups, successfully generating explant cultures or short-term culture of epithelial cells growing in 2D (Ethier et al. 1993; Speirs et al. 1998; Hass and Bertram 2009; Bruna et al. 2016).

For those scientists who are not embedded within research groups based at hospital sites, access to human tissue can be a problem. Additionally, the access and use is tightly regulated in some countries, which can present further obstacles. This was recognised by the UK charity Breast Cancer Now, who commissioned two gap analyses where clinical and scientific breast cancer experts discussed barriers in obtaining human breast tissue (Thompson et al. 2008; Eccles et al. 2013). As a direct result, a specialist breast cancer biobank was established, the Breast Cancer Now Tissue Bank (BCNTB; http://www.breastcancertissuebank.org). While a number of other breast biobanks exist worldwide (Wilson et al. 2015), the BCNTB is unique in that it offers a cell culture programme, which complements its routine collection of fresh frozen tumour and surrounding normal tissue, whole blood and serum samples, as well as formalin-fixed paraffin-embedded material. The BCNTB cell culture programme offers scientists a wide range of isolated purified cell populations, including explants, organoids, purified epithelial and myoepithelial cells and fibroblasts from different types of breast tumours. This provides scientists with new ways of modelling breast cancer without the need to use animals. Two good recent examples developed 3D models of the human breast duct with a view to using these to study ductal carcinoma in situ (DCIS), an early-stage, pre-invasive breast cancer. The introduction of mammographic screening in most Western nations has resulted in the increased detection of DCIS. This can be a precursor of invasive breast cancer in some women, but is an issue for doctors in terms of identifying who should receive treatment, which may turn out to be unnecessary in some cases, as not all DCIS will develop into invasive breast cancer (Marmot et al. 2013). Consequently, there is much interest in better understanding its biology, so a robust in vitro model is critical.

Two groups used cells from the BCNTB to develop physiologically relevant models to better understand the processes that underlie the transition of normal breast to DCIS and DCIS to invasive cancer. The first, a partially humanised 3D tri-culture model of normal breast, comprised luminal epithelial cell lines, primary human fibroblasts from the BCNTB and immortalised human myoepithelial cells growing in 3D in a collagen I matrix (Nash et al. 2015). More recently, a fully humanised 3D in vitro model using material from the BCNTB was developed to study the relationship between luminal and myoepithelial cells, the disruption of which is a critical first step towards the development of DCIS into an invasive phenotype (Carter et al. 2017). These models are important, as research into the biology of DCIS has previously relied on animal models, notably the MIND model, which involves intra-ductal transplantation of either DCIS-like cell lines or fragments of xenografts derived from human DCIS into immunocompromised mice to functionally test molecular events occurring in the initial changes in premalignant progression (Behbod et al. 2009). With increased uptake of the use of the BCNTB cell culture programme by the research community, it is highly likely that additional humanised models will be developed to help scientists work towards reducing reliance on the use of animal models in biomedical research.

## Patient-derived organoids

Further technical advances towards more advanced disease modelling is the development of the patient-derived organoid (PDO) model. Although organoid modelling per se is not new, the way this is now being applied to human tissues is opening up new opportunities to study and understand disease processes. Organoids are generated from small fragments of tissue from human tumours by mechanical and enzymatic disaggregation and plating in basement membrane extract, which can be maintained in culture. Because cells are maintained in 3D and retain critical cell–cell and cell–matrix interactions, these organoid models can be perceived as an intermediary between in vitro cell lines and animal xenograft models. While still a relatively new method, organoid cultures have enormous potential, with PDOs now derived from a number of different types of primary and metastatic human tumours with good success (van de Wetering et al. 2015; Bruna et al. 2016). By using patient tissue for research, the translational impact could increase greatly, with the possibility of advancing personalised medicine. Furthermore, this will certainly reduce, potentially even eliminating, the need for animals as pre-clinical models in the longer term. The use of patient tissue is possible through tissue banks such as the BCNTB mentioned above and others.

## Conclusions

Models to study breast cancer have evolved in the last few decades, gradually increasing in complexity to reflect native tissue architecture. Complementary to this, research is gradually moving away from 2D culture and in using animals to model breast cancer, towards developing humanised systems using human tissue samples from biobanks. In this era of precision medicine, this has real potential to revolutionise pre-clinical drug testing, offering an intermediate step, which could reduce or may even eventually replace the use of animals. Whilst it is unlikely that a single model alone will be used to recapitulate native tumour biology, using a combinatorial approach could impact on drug efficacy trials, improving translation into patients.
